# Pilot Assessment of Soil-Transmitted Helminthiasis in the Context of Transmission Assessment Surveys for Lymphatic Filariasis in Benin and Tonga

**DOI:** 10.1371/journal.pntd.0002708

**Published:** 2014-02-13

**Authors:** Brian K. Chu, Katherine Gass, Wilfrid Batcho, Malakai 'Ake, Améyo M. Dorkenoo, Elvire Adjinacou, 'Eva Mafi, David G. Addiss

**Affiliations:** 1 Neglected Tropical Diseases Support Center, Task Force for Global Health, Atlanta, Georgia, United States of America; 2 Programme National de Lutte Contre les Maladies Transmissibles, Ministère de la Santé, Cotonou, Benin; 3 Public Health Division, Ministry of Health, Nuku'alofa, Tonga; 4 Programme National d'Elimination de la Filariose Lymphatique, Ministère de la Santé, Lomé, Togo; 5 Children Without Worms, Task Force for Global Health, Atlanta, Georgia, United States of America; National Institute of Parasitic Diseases, Chinese Center for Diseases Control and Prevention, China

## Abstract

**Background:**

Mass drug administration (MDA) for lymphatic filariasis (LF) programs has delivered more than 2 billion treatments of albendazole, in combination with either ivermectin or diethylcarbamazine, to communities co-endemic for soil-transmitted helminthiasis (STH), reducing the prevalence of both diseases. A transmission assessment survey (TAS) is recommended to determine if MDA for LF can be stopped within an evaluation unit (EU) after at least five rounds of annual treatment. The TAS also provides an opportunity to simultaneously assess the impact of these MDAs on STH and to determine the frequency of school-based MDA for STH after community-wide MDA is no longer needed for LF.

**Methodology/Principal Findings:**

Pilot studies conducted in Benin and Tonga assessed the feasibility of a coordinated approach. Of the schools (clusters) selected for a TAS in each EU, a subset of 5 schools per STH ecological zone was randomly selected, according to World Health Organization (WHO) guidelines, for the coordinated survey. In Benin, 519 children were sampled in 5 schools and 22 (4.2%) had STH infection (*A. lumbricoides*, *T. trichiura*, or hookworm) detected using the Kato-Katz method. All infections were classified as light intensity under WHO criteria. In Tonga, 10 schools were chosen for the coordinated TAS and STH survey covering two ecological zones; 32 of 232 (13.8%) children were infected in Tongatapu and 82 of 320 (25.6%) in Vava'u and Ha'apai. All infections were light-intensity with the exception of one with moderate-intensity *T. trichiura*.

**Conclusions:**

Synchronous assessment of STH with TAS is feasible and provides a well-timed evaluation of infection prevalence to guide ongoing treatment decisions at a time when MDA for LF may be stopped. The coordinated field experiences in both countries also suggest potential time and cost savings. Refinement of a coordinated TAS and STH sampling methodology should be pursued, along with further validation of alternative quantitative diagnostic tests for STH that can be used with preserved stool specimens.

## Author Summary

Since 2000, some 2 billion preventive chemotherapy treatments have been delivered for lymphatic filariasis (LF), many in areas co-endemic for soil-transmitted helminthiasis (STH). A transmission assessment survey (TAS) is recommended to determine if such ‘mass drug administration (MDA)’ for LF can be stopped in a specified evaluation unit. The TAS also provides an opportune platform to simultaneously conduct STH assessments and make informed programmatic decisions about STH control. Through piloting this coordinated TAS and STH survey approach in Benin and Tonga, we found that after 5–6 rounds of annual MDA, STH prevalence was 4.2% in Benin and 13.8–25.6% in Tonga. All infections were light intensity except one moderate *A. lumbricoides* case in Tonga. Our study highlights that a coordinated strategy is operationally feasible, programmatically relevant, and potentially cost-effective. A more robust sampling methodology for coordinated TAS and STH surveys should be further explored, as well as alternative diagnostic tests for STH.

## Introduction

Soil-transmitted helminthiasis (STH) is a neglected tropical disease (NTD) that characteristically affects the poorest individuals in developing nations. Approximately 890 million children worldwide reside in STH-endemic areas and are at risk of STH-related anemia, malnutrition, and impaired physical and cognitive development [1]. To reduce this morbidity, the World Health Organization (WHO) recommends preventive chemotherapy (PC), specifically mass drug administration (MDA) of anthelminthic drugs to school-aged children and other at-risk individuals.

MDA is also a core strategy for controlling other NTDs, including lymphatic filariasis (LF). Since 2000, the Global Program to Eliminate Lymphatic Filariasis (GPELF) has delivered some 2 billion treatments of PC that includes albendazole donated by GlaxoSmithKline [2]. In addition to its role in mass treatment for LF, albendazole is active against a wide range of intestinal human parasites, including the STH-related group of *Ascaris lumbricoides*, *Trichuris trichiura*, and hookworm [3]. Given the large overlap between LF and STH-endemic areas and the effectiveness of albendazole, whether alone or in combination with ivermectin or diethylcarbamazine (DEC), in reducing intensity of STH infection [4]–[7], the impact of the LF program on STH is undoubtedly very significant. Few studies, however, have documented the impact of the LF program on STH, which is important now for three main reasons. First, several countries have completed five or more effective annual rounds of MDA for LF, and already may have interrupted LF transmission in some implementation units. They will soon be conducting the WHO-recommended transmission assessment survey (TAS) to assess whether MDA for LF can be stopped. Second, as countries discontinue community-wide MDA for LF, they will most likely transition to school-based PC for STH under current WHO guidelines. The recommended frequency of PC for school-aged children depends on the prevalence of STH, and assessing the new baselines of infection will guide treatment decisions for school-based PC. Finally, it is unclear whether focusing PC on school-age children after several years of community-wide deworming as an ancillary benefit of the LF program will be adequate to prevent recrudescence of STH in pre-school aged children and adults. To determine this, baseline assessments for STH will be required as MDAs for LF are discontinued.

The TAS is a comprehensive evaluation of the prevalence of filarial antigen (using the immunochromatographic (ICT) test (Alere Inc., Waltham, MA, USA) in areas endemic for *W. bancrofti*) or antibody (using the Brugia Rapid test [Reszon Diagnostics International, Subang Jaya, Selangor, Malaysia] in areas endemic for *Brugia spp.*) in the blood of a randomly selected sample of 6–7 year old children in a defined programmatic area. It is designed to allow for clear programmatic decisions regarding discontinuation of MDA for LF. The TAS may also be a very timely and suitable platform for collecting, in a coordinated manner, data critical for STH control.

Several issues must be considered when coordinating TAS and STH surveys. Programmatically, sampling strategies and survey procedures must be established with consideration of key criteria (Table 1). Epidemiologically, any STH assessment and resulting treatment guidelines must take into account the shift from community-wide MDA for LF to school-based MDA for STH. Finally, recommendations must also be cost-effective and feasible because in many situations, national deworming programs may not be established or prepared to implement post-LF treatment strategies for STH control.

**Table 1:** Comparison of TAS and STH survey criteria.

Characteristic	TAS (LF)^1^	STH survey^2^
Primary assessment	Stopping MDA, interruption of transmission	Determining MDA frequency
Evaluation area	Evaluation unit (one or more implementation units)	Ecologic zone(s)
Location	Schools if primary enrollment ≥75% Households if primary enrollment <75%	Schools
Target age group	6–7 year olds (1^st^–2^nd^ grade for school surveys)	3^rd^ grade children
Sampling strategy	LQAS^3^ (cluster or systematic survey), or census	Cluster survey
Child selection	Fixed proportion (sampling interval)	Fixed number per school
Sample size	Cluster: 759–1692, Systematic: 284–846	∼250 per ecological zone
Sample size incorporates measurement of error	Yes	No
Diagnostic specimen	Blood	Stool
Diagnostic tool	Immunochromatographic (ICT) test^4^	Kato-Katz
Programmatic decision criteria	Critical cutoff value	Point estimate

1 World Health Organization (2011) Global Programme to Eliminate Lymphatic Filariasis: Monitoring and epidemiological assessment of mass drug administration: a manual for national elimination programs. Geneva.

2 World Health Organization (2011) Helminth control in school-age children: a guide for managers of control programmes – 2nd ed. Geneva.

3 Lot quality assurance sampling.

4 For *W. bancrofti*.

To evaluate the potential of coordinated TAS and STH surveys in the context of these opportunities and challenges, pilot studies were conducted in TAS-eligible sites in Benin, in West Africa, and in the Pacific island-nation of Tonga. Lessons learned from these experiences may help identify best practices for conducting TAS and STH surveys together.

## Methods

### Site Selection

Selected study sites in Benin and Tonga are described in Table 2. In accordance with TAS eligibility guidelines, the study areas were designated as evaluation units (EU) meeting the criteria of having, i) at least five rounds of MDA with coverage ≥65% of the total population, ii) microfilaria (mf) prevalence rates <1% (or circulating filarial antigen (CFA) rates <2%) in all test sites, and iii) a population under two million [8].

**Table 2:** Evaluation unit characteristics for LF and STH assessment.

Country	Evaluation Unit	Population	Area (km^2^)	LF MDA history (*current status*)	LF MDA medicines	STH baseline prevalence in EU
**Benin**	Tchaourou (district)	143,108	7,256	6 rounds 2006–2011 (*not stopped MDA*)	Ivermectin+Albendazole^1^	0% (n = 50) *2009 survey of 1 school*
**Tonga**	Tongatapu, Vava'u, Ha'apai (island groups)	106,146	717	5 rounds 2001–2005 (*stopped MDA*)	DEC+Albendazole^2^	10.6% (n = 216) *2001–2002 survey of 2 schools*

1 Excludes children <5 years old and under 90 cm height.

2 Excludes children <2 years old.

In addition, EUs with differing geographical and epidemiological profiles were chosen to measure STH prevalence and the logistic capacity of combining surveys in varied settings. For Benin, the EU selected for the combined TAS and STH survey was the district of Tchaourou, which completed six rounds of LF MDA (ivermectin and albendazole), most recently in 2011. No other anthelminthic MDAs had been conducted in the area. In a 2009 national STH survey, no STH infections were identified among 50 children tested in one school in Tchaourou. The overall national STH prevalence was 5.2%, covering 757 children in 15 schools. In comparison, Tonga completed five rounds of LF MDA (DEC and albendazole) in 2005. Due to its small population, the entire country was designated as the EU, subdivided into the three major island groups of Tongatapu, Vava'u, and Ha'apai. A 2001–2002 Pacific Islands STH survey sampled 216 children in two schools (one urban and one rural) in Tonga; the STH prevalence in these children was 11.1%.

### Sampling Strategy

#### LF TAS

A guiding principle of incorporating STH assessment into the TAS was to make it as feasible as possible without interrupting TAS methods and survey requirements. STH survey and stool collection procedures were, therefore, built into the framework of the existing TAS protocol, which consists of rigorous sampling strategies dependent upon EU-specific factors such as the primary enrolment rate, target population size, total number of schools, and vector and filarial parasite species [8]. For Benin, the calculated TAS survey design was a cluster sample of 30 schools with a target sample size of 1552 first- and second-grade children. Schools and eligible children were randomly chosen based on systematic sampling procedures outlined in the TAS protocol. For the TAS in Tonga, a census of all estimated 3293 first graders in 127 schools was recommended to maintain consistency with the country's 2007 stop-MDA survey and regional LF elimination guidelines [9].

#### STH

STH was assessed in accordance with WHO guidelines for helminth control, which advocate sentinel site evaluation of third grade children in schools across a homogeneous ecological zone [10]. Because the currently recommended sample size for STH surveys is smaller than for TAS, a subset of five TAS schools per ecological zone was selected for STH assessment. In Tonga, the main island of Tongatapu was designated as one ecological zone and the smaller, more rural islands of Vava'u and Ha'apai a separate one, resulting in ten schools selected for the combined TAS and STH survey. In Benin, the entire EU of Tchaourou comprised a single ecological zone and, therefore, five schools were selected for the coordinated survey. All schools were chosen randomly with the exception of two schools in Tonga, which were pre-selected based on their having been included in the 2001–2002 STH survey.

Within each school, 50 third-grade children were then randomly chosen for stool collection; however, if the school selected for STH assessment did not have 50 third grade children, another grade of an older age group was sampled to give a total of at least 50 children per school. In Benin, an additional 50 TAS-eligible first and second graders were sampled per school to compare infection prevalence in this age group with that among third-graders and to also explore the benefits and limitations of collecting multiple specimens from the same group of children. A neighbouring school was sampled in case the selected school did not meet the required number of first and second, or third grade children. Table 3 summarizes the sampling strategy for both TAS and STH surveys in Benin and Tonga.

**Table 3:** Sampling strategy for coordinated TAS and STH surveys.

		LF (TAS)		STH survey		
Country	Evaluation unit	Survey design	Target sample size	Number of ecological zones in EU	Target number of schools surveyed^1^	Target sample size
**Benin**	Tchaourou (district)	Cluster survey of 30 schools	1,552 (1^st^–2^nd^ grade)	1	5	250 (1^st^–2^nd^ grade) 250 (3^rd^ grade)
**Tonga**	Tongatapu, Vava'u, Ha'apai (island groups)	Census of 127 schools	3,293 (1^st^ grade)	2	10	500 (3^rd^ grade)

1 5 schools per ecological zone; all schools randomly selected from TAS sample except two schools in Tonga that were included in 2001–2002 STH survey.

### Specimen Collection and Processing

All specimens were collected in the morning for TAS and STH surveys using separate three-person field teams that included a team leader and two laboratory technicians. With the assistance of schoolteachers, all eligible children from whom informed written and oral consent had been obtained were identified, lined up according to grade in school, and enumerated for random selection according to each survey's sampling protocol.

In both countries, all selected children had demographic data (i.e. name, age, and sex) recorded on data collection forms by the appropriate survey team. The LF team tested first and second grade children for *W. bancrofti* infection using ICT cards as per the TAS protocol. Third grade children were surveyed by the STH team at a different station and provided a single stool sample in a container marked with a unique identifier, which was subsequently logged and placed in cold chain storage. In Benin, first and second grade children selected for both surveys provided the stool sample before ICT testing. ICT cards were read and recorded at the schools while stool samples were transported to a nearby laboratory shortly after collection to be processed and evaluated for STH using a single Kato-Katz slide. The WHO-recommended Kato-Katz method is the most widely used diagnostic tool for sentinel site monitoring because it allows for quantification of egg density and does not require sophisticated laboratory equipment. Laboratory technicians on the STH field teams were trained in proper Kato-Katz technique [11], [12], especially the time-sensitivity of preparing and reading samples. As a result, all stool samples were evaluated within four hours of collection for *A. lumbricoides*, *T. trichiura*, and hookworm. Both infection prevalence and quantity of eggs for each species were recorded on data collection forms. The field team leader reviewed 10% of the daily Kato-Katz slides to further corroborate results.

### Data Collection and Analysis

TAS results were classified as *positive, negative, or invalid (i.e. test malfunction)* based on ICT card readings. STH parasitological results were classified as *positive* or *negative* based on detection or absence of helminth eggs of any species. Intensity of infection was quantified by the number of eggs per gram (epg) (egg count multiplied by 24) and then categorized as *light, moderate, or heavy* under standard threshold classification [10]. The geometric mean epg was calculated only for infected persons. Confidence intervals were calculated with 95% confidence level using the standard Wald asymptotic confidence limits. The chi-square test was used to determine statistical significance of STH infection prevalence by age group in Benin. Univariate analysis was conducted on infection prevalence and intensity with respect to age, sex, location, and helminth species.

### Ethics Statement

Ethical board approval was received by the Comité National Provisoire d'Ethique pour la Recherche en Santé (Benin) and within the Ministry of Health (Tonga) prior to the start of the survey. The respective Ministries of Education and selected school principals approved the study protocols.

In both Benin and Tonga, informed consent sheets with details of the study in local languages were distributed to children for their parent or guardian to sign in advance of the survey date. Only children who returned signed consent forms were eligible for selection. In addition, each selected child was required to give verbal assent to participate after receiving an oral explanation of the assessment procedures. Only assenting children were sampled and included in the surveys.

## Results

### Benin

The LF TAS and STH survey results are presented in Table 4. Of 1601 children sampled for TAS in 30 primary schools, none were positive by ICT test, including the 396 children surveyed in the five schools where both TAS and STH assessments were done (note: a sixth school was surveyed for STH due to insufficient sample size in one of the schools). In those schools, 519 children were examined for STH, resulting in a total prevalence of 4.2%, ranging from 0.0–10.0%.

**Table 4:** Benin TAS and STH survey results.

Survey (n schools)	n(+)/total	%	95% CI	Moderate or heavy infection %	Geometric mean eggs per gram (epg)^1^
TAS (30)	0/1601	0.0	-	-	-
STH (5)	22/519	4.2	(2.5–6.0)	0.0	-
*A. lumbricoides*	4/519	0.8	(0.0–1.5)	0.0	151.6
*T. trichiura*	1/519	0.2	(0.0–0.6)	0.0	72.0
Hookworm	15/519	2.9	(1.5–4.3)	0.0	91.5

1 Mean epg was only calculated for positive cases.

Table 4 also shows the prevalence and intensity of STH infection by species. Hookworm was the most prevalent (2.9%) infection while just minimal presence of *Ascaris lumbricoides* (0.8%) and *Trichuris trichiura* (0.2%) were detected. No mixed STH infections were found. The geometric mean epg was 151.6 for *A. lumbricoides*, 72.0 for *T. trichiura*, and 91.5 for hookworm, light-intensity infections as classified by WHO [10]. No moderate or heavy infections were found for any infection species.

Third grade children aged 8–9 had a higher prevalence of overall STH infection (4.9%) than first and second grade children aged 6–7 (2.7%), but the difference was not statistically significant (p = 0.19) (Table 5). A comparison of the two age groups by type of infection also yielded similar prevalence patterns and non-statistically significant differences.

**Table 5:** Benin STH infection by age group.

	Age 6–7			Age 8–9			
Infection	n(+)/total	%	95% CI	n(+)/total	%	95% CI	p-value
*A. lumbricoides*	1/256	0.4	(0–1.6)	3/263	1.1	(0.0–2.4)	0.62
*T. trichiura*	0/256	0.0	-	1/263	0.4	(0.0–1.1)	1.00
Hookworm	6/256	2.4	(0.5–4.2)	9/263	3.4	(1.2–5.6)	0.60
**Total**	**7/256**	**2.73**	**(0.7–4.7)**	**13/263**	**4.9**	**(2.3–7.6)**	**0.19**

### Tonga

A total of 2434 children were sampled for TAS in 127 primary schools, resulting in 7 ICT positive tests (0.29%) (Table 6). Ten of those schools (5 per ecological zone) also were surveyed for STH and infection prevalence was 13.8% in Tongatapu and 25.6% in Vava'u/Ha'apai. Eight of the ten schools had at least one STH positive child with an overall range of 0.0–47.9%.

**Table 6:** Tonga TAS and STH survey results.

	LF (TAS)			STH			
Ecological zone	n schools	n children (+)/total	%	n schools	n children (+)/total	%	95% CI
Tongatapu	74	3/1800	0.2	5	32/232	13.8	(9.3–18.2)
Vava'u/Ha'apai	53	4/634	0.6	5	82/320	25.6	(21.0–34.9)
**Total** 1	**127**	**7/2434**	**0.3**	**-**	**-**	**-**	**-**

1 Totals were not calculated for STH because it was a cluster design survey for each ecological zone and not the entire country.

Table 7 highlights the prevalence and intensity of STH infection by species in each ecological zone. In Tongatapu, *T trichiura* was the most prevalent infection (7.8%) followed by *A. lumbricoides* (6.9%) and hookworm (3.0%). All infections were classified as light infection with a geometric mean epg of 184.6 for *A. lumbricoides*, 85.5 for *T. trichiura*, and 74.6 for hookworm.

**Table 7:** Tonga STH infection species and intensity.

Ecological zone (n schools)	Infection	n(+)/total	%	95% CI	Moderate/heavy infection %	Geometric mean eggs per gram (epg)^1^
Tongatapu (5)	*A. lumbricoides*	16/232	6.9	(3.6–10.2)	0.0	184.6
	*T. trichiura*	18/232	7.8	(4.3–11.2)	0.0	85.5
	Hookworm	7/232	3.0	(0.8–5.2)	0.0	74.6
Vava'u/Ha'apai (5)	*A. lumbricoides*	70/320	21.9	(17.3–26.4)	1.4^2^	248.1
	*T. trichiura*	13/320	4.1	(1.9–6.2)	0.0	44.3
	Hookworm	13/320	4.1	(1.9–6.2)	0.0	137.4

1 Mean epg was only calculated for positive cases.

2 One *A. lumbricoides* infection was moderate intensity with 28,824 epg.

In Vava'u and Ha'apai, *A. lumbricoides* was the most prevalent infection (21.9%) followed by *T. trichiura* (4.1%) and hookworm (4.1%).. All were classified as light intensity except one moderate-intensity *A. lumbricoides* infection (28,824 epg) on Ha'apai. The geometric mean epg was 248.1 for *A. lumbricoides*, 44.3 for *T. trichiura*, and 137.4 for hookworm.

## Discussion

Recent years have seen a notable trend towards integrating NTD control activities such as mapping and monitoring and evaluation [13]–[15]. From a public health policy standpoint, integrated NTD activities can result in improved cost-effectiveness and resource efficiency, particularly where endemic populations overlap and drug treatments are similar, as is the case with LF and STH. Combining STH surveys with TAS is relevant programmatically in that the potential cessation of MDA for LF has direct implications for transitioning to STH treatments. The aim of these pilot studies in Benin and Tonga, therefore, was not only to provide guidance for STH treatment, but also to assess the practical and methodologic challenges of a coordinated approach to TAS and STH surveys. The intent was to raise issues to be considered in the shift from community-wide MDA for LF to school-based drug distribution for STH.

The TAS results indicated that LF prevalence was below the presumed critical threshold for transmission in both EUs and that MDA for LF can be discontinued. In contrast, STH remained prevalent, even after 5–6 years of community-wide MDA. The coordinated assessment provided limited insight into the impact of LF MDA on STH. The near absence of moderate or heavy infections is consistent with a positive impact, although several factors, including drug coverage, treatment regimen, and access to sanitation could have influenced the results. In particular, the treatment regimen of ivermectin (with albendazole) in Benin may have led to greater impact on STH than in Tonga where individuals were treated with DEC and albendazole [16], [17]. Baseline STH data in both countries were also deemed insufficient to make statistically valid comparisons with post-MDA STH levels. The absence of adequate baseline data on STH is to be expected in other settings that are eligible for the TAS, as many LF elimination programs were begun before the move towards integrated NTD programming. Based on current WHO guidelines for settings in which 5–6 years of MDA for LF have been completed [10], bi-annual school-based treatment for STH would be recommended in Benin where STH prevalence was 4.2%. Applying these same guidelines to Tonga, once- and twice-yearly treatment would be recommended for the ecological zones with 13.8% and 25.6% prevalence, respectively.

Coordinated TAS and STH assessments provide a timely opportunity to make informed programmatic decisions about STH control. Synchronous assessment approximately 6–12 months after the previous MDA is recommended for TAS and is ideal for assessing STH. Over longer periods, STH prevalence may return to pre-treatment levels as a result of reinfection [15]. The 6-year interval between the last MDA in Tonga (2005) and the current assessment (2011) may have contributed to an apparent recrudescence of STH prevalence and the presence of moderate-intensity *A. lumbricoides* infection. Such a prolonged interval also complicates the application of the WHO guidelines for school-based anthelminthic treatment [10]. After 5–6 years of MDA, WHO recommends continuing annual school-based mass treatment if the STH prevalence is ≥10% and <20%, whereas no mass treatment is recommended if this prevalence is observed at baseline, i.e., in the absence of MDA. Synchronous assessment of STH during a TAS both simplifies implementation of these guidelines and minimizes risk of recrudescence in the evaluation area.

There are logistical benefits to coordinating STH surveys with TAS. Most notably, transportation time and cost can be reduced as a result of fewer visits to the same schools. In Benin, the time required to complete both surveys was reduced by an estimated 50% by implementing them together. When applied to larger TAS EUs or remote geographical areas, these savings would likely be even greater. In the outer Tongan islands of Ha'apai and Vava'u for instance, approximately two weeks would have been required for the STH survey alone if not carried out through a coordinated approach with the TAS. Both in Benin and in Tonga, separate three-person field teams were devoted to LF and STH surveys, so comparisons between single and multiple team approaches could not be directly measured. Given proper training and organization, however, a more integrated team with fewer technicians would be possible, which would further decrease costs (e.g., in reducing the number of vehicles required) and enhancing NTD program integration. Data on cost were not rigorously collected, a limitation of this study. Even if the cost savings of synchronous assessment are not substantial, the operational feasibility and the opportunity to assess STH prevalence before recrudescence potentially occurs are strong arguments for conducting STH surveys during TAS. Coordinated surveys in Benin and Tonga were also programmatically efficient; fewer schools needed to be organized and all informed consent procedures were arranged together. This likely minimized refusals and promoted understanding of the health benefits of both LF elimination and STH control.

The field experiences in Benin and Tonga also highlighted certain challenges with coordinating TAS and STH surveys. Perhaps the biggest logistical challenge arose from the time sensitivity of the recommended Kato-Katz method for processing and examining stool samples for STH. Specimens generally must be examined microscopically within 24 hours of collection, and within 4 hours for ideal hookworm detection [18]. This constraint necessitates either same-day transportation of specimens to a nearby central laboratory or adding trained technicians to the TAS field team and examining specimens in the field. Both options may be cost-and/or time-prohibitive or altogether unfeasible. In Benin, skilled microscopists were available in a central laboratory, but transportation time to the laboratory from each school remained a challenge. In Tonga, laboratory space in Vava'u and Haa'pai was sometimes inadequate and a trained microscopist was consulted to lead survey efforts and train local staff. In this study, only one Kato-Katz slide was examined and logistic constraints could be greater if multiple slides were to be evaluated. Several of these challenges could be surmounted with an alternative to the Kato-Katz method in which specimens could be collected and preserved in the field and examined later in a central laboratory. Ideally, a quantitative assessment of infection intensity could be made by measuring a standard amount of stool (e.g. one gram) into a measured amount of preservative (e.g. formalin-ether or sodium acetate-acetic acid-formalin [SAF]). Several such methods are currently under evaluation and while preliminary results for the FLOTAC [19], [20] and mini-FLOTAC [21] techniques have been promising, none, as yet, have been recommended by WHO. The mini-FLOTAC test may also be more cost-effective when compared to the Kato-Katz or FLOTAC methods [22], [23]. Further validation and standardization are needed in larger-scale studies in varied epidemiologic settings [20], [24]–[26].

These pilot studies in Benin and Tonga serve as starting points for exploring standard sampling strategies and operating procedures for coordinated TAS and STH surveys. Key considerations include the target age group; the number of children to be sampled for the STH assessment and the method for selecting them; and the geographic and programmatic implications of the LF EU for STH control. WHO TAS guidelines recommend testing 6–7 year old children for LF (i.e., school children in first and second grades) [8] while STH guidelines recommend testing third grade children (mostly 8–9 years old in Benin) in school-based surveys [10]. Both age groups were assessed for STH in Benin, and no significant difference in infection prevalence was observed, despite the older group being born before the start of MDA (2005) and having received more rounds of treatment (as ivermectin is distributed to children ≥5 years and ≥90 cm height). While these data are limited, they suggest that sampling either age group for STH would provide similar estimates of prevalence and intensity [27]. Some field teams may find it easier, in terms of logistics and organization, to collect blood from 8–9 year-old children for LF testing and stool samples from younger children in the same school for STH, as was done in Tonga. This approach avoids the need to determine which 6–7 year old children will be tested for LF alone and which will also be tested for STH (as was done in Benin) and may reduce the time required by the field team at each school. Further, it is generally less demanding on children to provide only a single specimen. On the other hand, it may be administratively and logistically easier in some settings to collect both specimens from the same children.

In both Tonga and Benin, survey teams followed the recommended WHO STH guidelines of testing approximately 250 children per ecological zone [10]. This approach provides a point estimate of STH prevalence and does not explicitly take into consideration uncertainty around that point estimate, but it does limit stool collection to five schools. Adopting the TAS sampling approach for STH assessment would require collecting stool specimens from children in all schools selected for the TAS, but would greatly reduce the number of specimens collected in each school. Even if the same total number of specimens were similar (e.g., ∼250), this approach would provide a more representative sample for STH within a given EU and would account for the design effect of cluster-based surveys. The operational feasibility of testing fewer children in a larger number of schools would be enhanced with a diagnostic method other than Kato-Katz, which can be used on preserved stool.

A closer alignment of STH and TAS methodologies also would also increase the efficiency of coordinated assessments. A TAS design for STH would eliminate the need to subset ecological zones or school selection for the STH survey. Within schools, the TAS systematic sampling approach was used effectively in this study to randomly select children for both LF and STH assessments. Team members were already familiar with the TAS methodology and did not have to learn or implement a secondary sampling procedure. A standardized STH methodology that leverages the robustness of the TAS strategy should be further examined and validated for future coordinated assessments.

Ecological zones, recommended as the basis for STH sampling [10] usually are considered to be larger than TAS EUs. Spatial homogeneity of STH within each zone is assumed, although this varies by helminth species. In particular, *A. lumbricoides* and *T. trichiura* may have a more focal distribution [28], [29], somewhat comparable to that of LF, which is generally considered a focal infection. Evidence is emerging that STH spatial heterogeneity may be considerably greater in post-treatment and urban settings. Use of the TAS methodology to assess STH prevalence and intensity within a specific administrative unit, where decisions regarding treatment for STH and other NTDs will be made and implemented, offers certain programmatic and epidemiologic advantages over the more general ecological approach, especially in post-treatment and urban areas.

Concomitant assessment for STH in the LF TAS raises several questions for further research. The implications of transitioning from community-wide MDA for LF to school-based treatment for STH control are unclear. It is possible that STH recrudescence will occur in adults and younger children in such settings. Access to clean water and sanitation is closely linked to sustained reductions in STH, and may need to be considered in guidelines for STH treatment in the post-LF settings [30], [31]. Alternative strategies to post-LF MDA assessment may provide complementary methods or measures for evaluating the impact on STH [32]. Finally, the cost-effectiveness of combining TAS and STH surveys should be more thoroughly assessed.

The intent of these pilot studies in Benin and Tonga was to evaluate the feasibility of coordinated TAS and STH surveys and to explore possible barriers to coordinated assessment. Despite the limited cost data acquired in this study, coordinated assessment appears highly feasible and potentially cost-saving when compared to stand-alone STH surveys. The greatest barrier to assessing STH within the TAS framework was the need to examine unpreserved stool within hours of collection using the recommended Kato Katz technique. Collection and preservation of stool specimens for later examination in a central laboratory could significantly enhance the feasibility and efficiency of combined assessment. Programmatically, determining the prevalence of STH at the point when LF MDA may be stopped is important for rational STH control. Coordinated TAS and STH surveys provide an integrated opportunity to make this assessment using the robust TAS methodology. Standardized guidelines should be developed and field-tested to identify the ideal sampling strategy, diagnostic tests, and specimen collection procedures.

## Supporting Information

Checklist S1 STROBE checklist.
(PDF)
